# Pazopanib for treating rhabdomyosarcoma in adult patients with poor performance status: A case report

**DOI:** 10.1111/1759-7714.14669

**Published:** 2022-09-21

**Authors:** Yuuya Nishii, Jun Sasaki, Misa Sudou, Ryo Yano, Saeko Tokisawa, Reiko Takaki, Takaaki Tokito, Tomoaki Hoshino

**Affiliations:** ^1^ Division of Respirology, Neurology, and Rheumatology, Department of Internal Medicine Kurume University School of Medicine Kurume Japan

**Keywords:** pazopanib, poor performance status, rhabdomyosarcoma, soft tissue sarcoma

## Abstract

Rhabdomyosarcoma (RMS) is a common soft tissue sarcoma usually observed in children. However, RMS rarely occurs in adults. The prognosis of adult RMS is poor and a standard chemotherapy regimen has not yet been established. Herein, we report the case of a 60‐year‐old Japanese woman with primary anterior mediastinal alveolar RMS (T3N0M0, stage III). The tumor increased aggressively despite first‐line treatment with doxorubicin (60 mg/m^2^ every 3 weeks for 1 cycle) and second‐line treatment with eribulin (1.4 mg/m^2^ every 3 weeks for 2 cycles). Although her shortness of breath and chest tightness worsened as the tumor compressed her heart and left main bronchus, and her performance status (PS) decreased to 3, third‐line treatment with pazopanib (800 mg once daily) was commenced. The treatment led to suppression of tumor growth and resulted in 4‐month progression‐free survival. Therefore, in cases of adult RMS, considering pazopanib treatment as an option may be beneficial, even with previous ineffective treatments or poor PS.

## Graphical abstract

Rhabdomyosarcoma (RMS) is rarely observed in adults and the prognosis is very poor. Despite previous ineffective therapy and poor performance status (PS), treatment with pazopanib suppressed aggressive tumor growth in the case reported here. Pazopanib treatment should be considered as a viable treatment option in cases of adult RMS with poor PS.

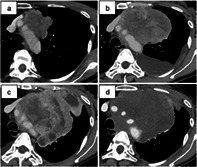

## INTRODUCTION

Rhabdomyosarcoma (RMS) is a type of soft tissue sarcoma (STS), observed mostly in children and rarely in adults. STS accounts for 1% of all adult cancers.^1^, ^2^ RMS accounts for only 3.3% of adult STS.^3^, ^4^

First‐line treatment comprises surgery in patients with early‐stage RMS^5^ and chemotherapy in unresectable cases. Doxorubicin is a widely accepted first‐line therapy;^6^ however, subsequent treatment strategies have not been standardized and prognosis of RMS is poorer in adults than in children.^4^, ^7^ Pazopanib, a multitargeted tyrosine kinase inhibitor, primarily targets vascular endothelial growth factor receptors 1–3, platelet‐derived growth factor receptors, fibroblast growth factor receptors, and c‐kit. In addition, pazopanib is effective in patients with STS previously treated with standard chemotherapy regimens.^8^ Herein, we report a case of mediastinal RMS treated with pazopanib after undergoing two ineffective regimens of doxorubicin and eribulin.

## CASE REPORT

**FIGURE 1:**
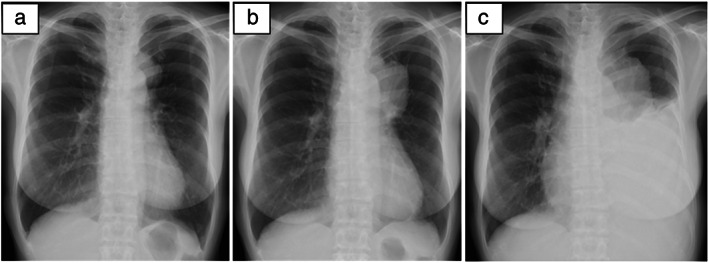
Chest X‐ray images. Images showing (a) the mediastinal tumor at the initial visit to our hospital, (b) one month after the initial visit, and (c) two months after the initial visit, at first‐line chemotherapy initiation

**FIGURE 2:**
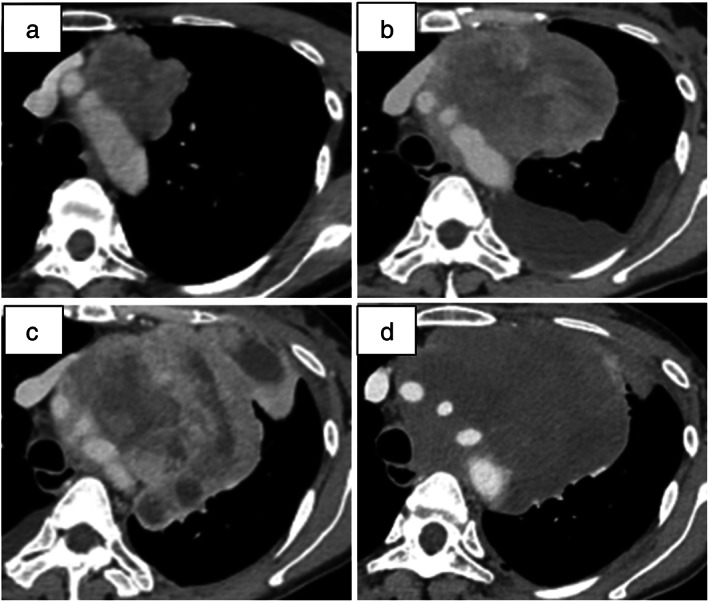
Contrast‐enhanced chest computed tomography images. Images showing the (a) anterior mediastinal tumor at the initial visit to our hospital and (b) two months after the first visit to our hospital, at first‐line chemotherapy initiation. (c) Image showing an aggressively increased tumor at the start of pazopanib treatment. (d) Image showing tumor size reduction and tumor contrast effect at 3 months after pazopanib initiation

**FIGURE 3:**
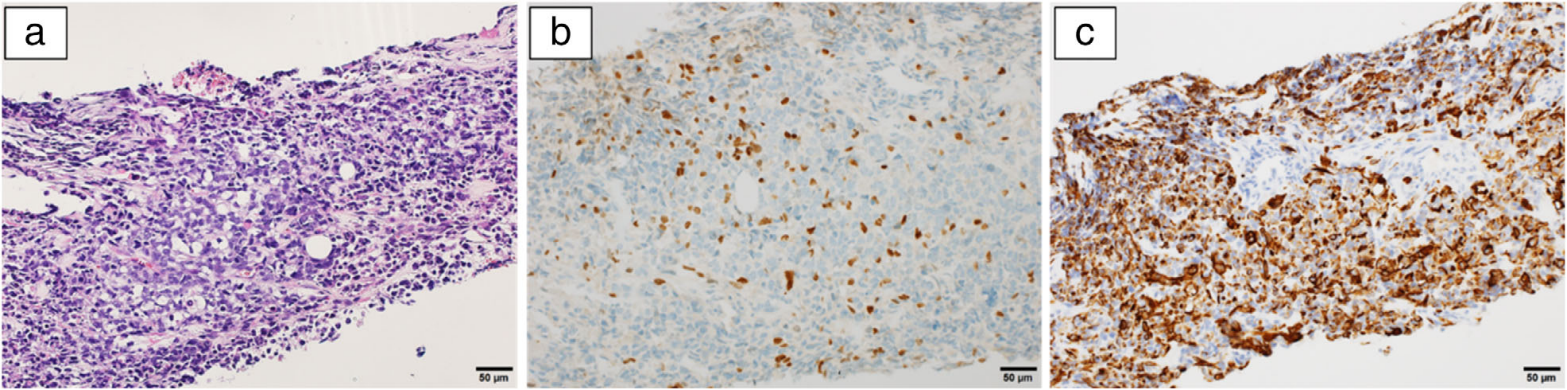
Histopathological examinations. (a) Hematoxylin and eosin staining revealed mesenchymal tumor cells with hyperchromatic nuclei proliferated in a solid alveolar pattern. (magnification, ×200). (b) Immunostaining revealed that the tumor cells were positive for myogenin (magnification, ×200), and (c) desmin (magnification, ×200)

A 60‐year‐old Japanese woman experienced hoarseness and was diagnosed with left‐sided recurrent nerve paralysis. Chest radiography revealed a mediastinal tumor (Figure 1a). Computed tomography (CT) revealed an anterior mediastinal tumor (size, 6.8 × 6.0 × 5.7 cm^3^) without calcification or obvious fat components (Figure 2a). Magnetic resonance imaging revealed tumor signals similar to that of the surrounding muscle tissue on fat‐suppression T1‐weighted imaging and unevenly high‐intensity signals on fat‐suppression T2‐weighted imaging. Histopathological examination of a CT‐guided biopsy specimen confirmed the diagnosis of a malignant alveolar tumor. The tumor cells were positive for cytokeratin AE1/AE3, vimentin, CD56, myogenin, desmin, myoD1, and HHF35, and negative for S100, p40, and myoglobin (Figure 3a–c).

Therefore, the patient was diagnosed with RMS (T3N0M0 stage III). However, the tumor had invaded the aorta and was unresectable. She was administered standard chemotherapy, doxorubicin (60 mg/m^2^ every 3 weeks) as a first‐line regimen. On treatment initiation, her performance status (PS) was 1. Furthermore, the patient had dyspnea on exertion and chest tightness due to aggressive tumor progression over 2 months after her initial hospital visit (Figures 1b,c and 2b). After one cycle of doxorubicin, her condition deteriorated and the tumor continued to grow rapidly. Although her PS had decreased to 2, she received second‐line therapy with intravenous eribulin (1.4 mg/m^2^ every 3 weeks). After two cycles of eribulin treatment, the patient experienced an adverse event (AE) of grade 3 neutropenia. Her dyspnea remarkably worsened. Moreover, the primary tumor grew further, compressing the heart, left main bronchus, and pulmonary artery (Figure 2c).

Although her PS further decreased to 3, she received third‐line therapy with pazopanib (800 mg daily). After pazopanib initiation, her shortness of breath slightly improved. Enhanced CT showed a slight decrease in tumor size and contrast effect inside the tumor (Figure 2d). Furthermore, she experienced no AEs (accordingly, no discontinuation or dose reduction) and was discharged from the hospital. Pazopanib treatment effectively suppressed disease progression for 4 months. However, the disease progressed and she died 7 months after first‐line treatment initiation.

## DISCUSSION

The objective response rate (ORR) and progression‐free survival (PFS) are poor in patients with RMS undergoing primary treatment of surgery for early‐stage cases or chemotherapy for unresectable cases. The five‐year overall survival (OS) in adult RMS, including resectable cases, is 21%, which is far poorer than that in pediatric RMS.^9^

Although a combination of vincristine, actinomycin D, and cyclophosphamide is well established for RMS in children, doxorubicin is a widely accepted first‐line therapy for STS,^6^ which agrees with the Japanese guidelines. Regarding second‐ or later‐line therapies for STS, three regimens of pazopanib, eribulin, and trabectedin have been reported as effective. However, which treatment supersedes the others remains unknown. The treatment response varies according to the histological type of STS, and limited data are available for RMS due to its rarity. In the present case, although the median PFS for STS is 5.5 months, first‐line doxorubicin was ineffective as only 1 month of PFS was observed.^10^

In a randomized phase III trial study comparing eribulin and dacarbazine for previously treated liposarcoma or leiomyosarcoma, eribulin significantly prolonged the OS. However, no significant differences in PFS (2.6 months) and ORR (4%) were observed.^11^ Moreover, a phase II trial conducted in Japan showed the efficacy of eribulin in liposarcoma and other types of advanced STS, including two RMS cases (3.9%), with a median PFS of 4.1 months and ORR of 0%. However, 96% of patients experienced grade 3–4 treatment‐related AEs, with neutropenia (86%) being the most frequently reported AE.^12^ In contrast, in the case reported here, we observed a very short PFS of only 1 month during second‐line treatment with eribulin, suggesting its ineffectiveness in this aggressive form of rare adult RMS.

In a phase III trial study of pazopanib in patients with metastatic STS and PS of 0–1, pazopanib significantly prolonged the median PFS compared with that with placebo (4.6 vs. 1.6 months, *p* < 0.0001), with few AEs above grade 3.^8^ In the present case, pazopanib treatment suppressed aggressive tumor growth, without any AEs and contributed towards 4 months of PFS, despite the ineffectiveness of previous treatment and her poor PS (3). Enhanced CT showed a decrease in tumor size and contrast effect inside the tumor after treatment with pazopanib. Therefore, we considered that the antitumor effects of pazopanib were mainly due to suppression of angiogenesis by targeting vascular endothelial growth factor receptors 1–3.

In conclusion, pazopanib treatment should be considered as a viable treatment option in patients with RMS and poor PS. Furthermore, we consider this case report to be helpful in formulating a treatment strategy for extremely rare cases of adult RMS with poor PS.

## CONFLICT OF INTEREST

The authors declare that they have no conflict of interest.
